# Overall and Cancer-Specific Survival in Patients With Renal Pelvic Transitional Cell Carcinoma: A Population-Based Study

**DOI:** 10.3389/fmed.2021.719800

**Published:** 2022-01-03

**Authors:** Tingting Hu, Shengjie You

**Affiliations:** ^1^Department of Chemoradiation Oncology, The First Affiliated Hospital of Wenzhou Medical University, Wenzhou, China; ^2^Department of Urinary Surgery, The People's Hospital of Lishui, Lishui, China

**Keywords:** upper tract urothelial carcinoma, renal pelvic transitional cell carcinoma, overall survival, cancer-specific survival, nomogram

## Abstract

**Background:** Renal pelvic transitional cell carcinoma (TCC) is a relatively rare tumor. This study aimed to develop two prognostic nomograms to predict overall survival (OS) and cancer-specific survival (CSS) in renal pelvic TCC patients.

**Methods:** Clinicopathological and follow-up data of renal pelvic TCC patients diagnosed between 2010 and 2015 were retrieved from the Surveillance, Epidemiology, and End Result (SEER) database. Univariate and multivariate Cox regression analyses were used to screen the independently prognostic factors. These independently prognostic factors were then utilized to build nomograms for predicting 3-, 4-, and 5- years OS and CSS of patients with renal pelvic TCC. The nomograms were assessed by calibration curve, receiver operating characteristic (ROC) curve and decision curve analysis (DCA).

**Results:** A total of 1,979 renal pelvic TCC patients were enrolled. Age, tumor size, histological type, American Joint Committee on Cancer (AJCC) stage, surgery, chemotherapy, bone metastasis and liver metastasis were confirmed as independently prognostic factors for both OS and CSS. The areas under the ROC curves (AUCs) of OS nomogram at 3-, 4- and 5-years in the training cohort were 0.797, 0.781, and 0.772, respectively, and the corresponding AUCs in the validation cohort were 0.813, 0.797, and 0.759, respectively. The corresponding AUCs of CSS nomogram were all higher than 0.800. The calibration curves and DCA indicated that both nomograms had favorable performance. Subgroup analyses showed that both nomograms perform in well and poorly differentiated patients.

**Conclusion:** In conclusion, we successfully developed and validated two valuable nomograms to predict the OS and CSS for renal pelvic TCC patients. The nomograms incorporating various clinicopathological indicators can provide accurate prognostic assessment for patients and help clinicians to select appropriate treatment strategies.

## Introduction

Upper tract urothelial carcinoma (UTUC) is a tumor that occurs in the renal pelvic and ureter, with an estimated incidence of 1–4 cases per 1,00,000 people per year (1). UTUC is characterized by aggressive behavior and ~25% of patients have regional metastases (2). In UTUC, renal pelvic tumors are more common than ureteral tumors, and the majority of renal pelvic tumors are transitional cell carcinoma (TCC) (3, 4), accounting for about 90% of all renal pelvic tumors (5). Renal pelvic TCC is a malignant tumor with the potential of multicentric origin (6). The distribution of left and right kidneys is roughly equal, and bilateral tumors are not common (6). Although many renal pelvic tumors were diagnosed early due to the development of novel technologies, the prognosis of advanced tumors is still poor, with the 5-year CSS is <50 and 10% for pT2/pT3 and pT4 patients, respectively (7, 8).

Although the traditional American Joint Committee on Cancer (AJCC) stage system is widely recognized as a robust prognostic prediction and stratification tools for cancer patients, it does not adequately cover patients' status and treatment information (9). A large number of studies had confirmed the limitations of the AJCC stage system and the superiority of comprehensive nomogram (10–12). Although several prognostic models had been developed for UTUC patients (13–15), it is worth noting that these studies consider renal pelvic tumors and ureteral tumors as one integral group, ignoring that they are not completely homogeneous in biology, and they may behave differently (16, 17). Therefore, it is very important to establish prognostic models for predicting the prognosis of patients with renal pelvic TCC. In previous studies, many risk factors and prognostic variables were identified for renal pelvic TCC patients, including age, grade, stage, surgery, chemotherapy, lymphatic invasion and tumor structure (6, 18–20). Unfortunately, to our knowledge, there is no thorough research focused on developing the prognostic prediction tools for renal pelvic TCC, which means that the probability of the outcome cannot be quantified.

The nomogram is a convenient prediction tool, which accurately predicts individual outcome and has been used to assess the prognosis of several cancer patients (21). In the present study, we aimed to develop two nomograms for predicting the overall survival (OS) and cancer-specific survival (CSS) of renal pelvic TCC patients based on the Surveillance, Epidemiology, and End Results (SEER) database.

## Materials and Methods

### Study Population Selection

Patients' data were downloaded from the SEER database using the SEER^*^Stat software (version 8.3.6). Patients diagnosed with renal pelvic TCC (ICD-O-3: 8120 and 8130) between 2010 and 2015 were included in this study. The exclusion criteria were as follows: (1) renal pelvic TCC is not the first primary tumor; (2) died but the cause of death is unclear; (3) unknown information, including age, tumor size, race, sex, grade, histological type, AJCC TNM stage, surgery, radiotherapy, chemotherapy, and metastatic information.

**Figure 1:**
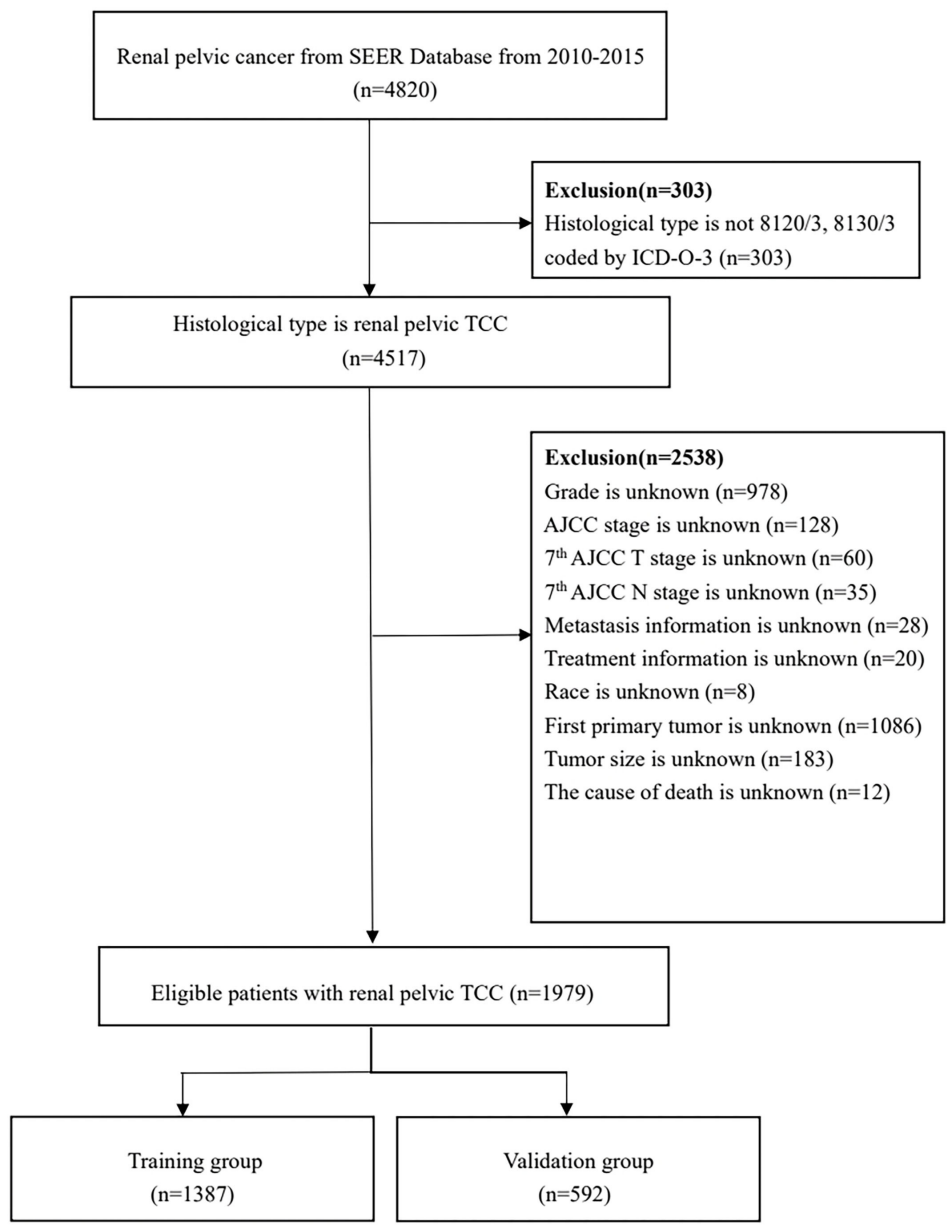
Flowchart of patients identified in this study. SEER, Surveillance, Epidemiology, and End Result; TCC, transitional cell carcinoma; AJCC, American Joint Committee on Cancer.

Ultimately, a total of 1,979 patients were enrolled and randomly divided into the training and validation cohorts with a ratio of 7:3 (22). Investigating prognostic factors independently affecting OS and CSS and developing prognostic nomograms were performed in the training cohort, and the nomograms were externally validated in the validation cohort. The detailed process for patients screening is presented in Figure 1.

### Data Collection

Variables included in the present study were demographic, cancer, treatment and metastatic data. Demographic variables included age, race, and sex. The cancer characteristics included tumor size, grade, histological type, and AJCC TNM stage. Treatment characteristics included surgery, chemotherapy and radiotherapy. Metastatic data included bone metastasis, brain metastasis, liver metastasis, and lung metastasis. In the SEER database, age and tumor size were recorded as continuous variables, but in our study, X-tile software (Yale University, New Haven, Connecticut, USA) was used to confirm the optimal cutoff values of age and tumor size in both OS and CSS (23). The best cutoff values of age for OS were 71- and 83-years, the best cutoff values of age for CSS were 68- and 83-years, and the best cutoff values of tumor size for OS and CSS were both 45 and 67 mm.

### Statistical Analysis

SPSS 25.0 (IBM) and R software (version 3.6.1) were performed for all statistical analyses. *P* value < 0.05 (two sided) was considered as a statistically significant cutoff value. Univariate and multivariate Cox regression analyses were applied to determine the independent prognostic factors. The time-dependent receiver operating characteristic (ROC) curves of the prognostic nomograms were generated (24). The areas under the ROC curves (AUCs) were used to show the discrimination of nomogram, and were further compared with the AUCs of the all independently prognostic factors. Moreover, the calibration curves were established to compare the consistency between nomogram-predicted and actual outcomes. The range of threshold probabilities and the magnitude of benefit were identified by decision curve analysis (DCA) (25). Furthermore, patients in the training cohort and validation cohort were divided into high-risk, middle-risk and low-risk groups according to the X-tile determined cutoff values (23), and the Kaplan-Meier (K-M) curves and log-rank test were then generated (26). Finally, subgroup analysis was performed and the performance of nomograms in well differentiated and poorly differentiated groups were evaluated with ROC and K-M curves.

## Results

### Patients Baseline Characteristics

The baseline information of 1,979 renal pelvic TCC patients are shown in Table 1. The mean age of all patients was 70.7 ± 11.2 years old, and 1,714 (86.6%) were white race. The majority of grade is IV (63.3%), while 91.8% were in M0 stage. Although the lung metastasis is the most common pattern for renal pelvic TCC, the incidence is lower than 3.5% of the cases. For the treatment status, most patients underwent surgery but without radiotherapy.

**Table 1:** Clinical and pathological features of patients diagnosed with renal pelvic TCC.

**Characteristics**	**Total cohort** **(***n*** = 1,979)**	**Training cohort** **(***n*** = 1,387)**	**Validation cohort** **(***n*** = 592)**
Age, years	70.7 ± 11.2	70.9 ± 11.4	70.2 ± 10.9
Tumor size, mm	46.7 ± 32.2	46.4 ± 26.6	47.4 ± 42.5
**Race**			
White	1,714 (86.6%)	1,201 (86.6%)	513 (86.7%)
Black	98 (5.0%)	68 (4.9%)	30 (5.1%)
Other	167 (8.4%)	118 (8.5%)	49 (8.3%)
**Sex**			
Female	1,030 (52.0%)	781 (56.3%)	249 (42.1%)
Male	949 (48.0%)	606 (43.7%)	343 (57.9%)
**Grade**			
I	67 (3.4%)	41 (3.0%)	26 (4.4%)
II	226 (11.4%)	168 (12.1%)	58 (9.8%)
III	434 (21.9%)	294 (21.2%)	140 (23.6%)
IV	1,252 (63.3%)	884 (63.7%)	368 (62.2%)
**Histological type**			
TCC, NOS	889 (44.9%)	631 (45.5%)	258 (43.6%)
Papillary TCC	1,090 (55.1%)	756 (54.5%)	334 (56.4%)
**AJCC**			
I	560 (28.3%)	388 (28.0%)	172 (29.1%)
II	187 (9.4%)	143 (10.3%)	44 (7.4%)
III	725 (36.6%)	491 (35.4%)	234 (39.5%)
IV	507 (25.6%)	365 (26.3%)	142 (24.0%)
**T stage**			
T1	597 (30.2%)	418 (30.1%)	179 (30.2%)
T2	213 (10.8%)	163 (11.8%)	50 (8.4%)
T3	904 (45.7%)	624 (45.0%)	280 (47.3%)
T4	265 (13.4%)	182 (13.1%)	83 (14.0%)
**N stage**			
N0	1,638 (82.8%)	1,142 (82.3%)	496 (83.8%)
N1	168 (8.5%)	125 (9.0%)	43 (7.3%)
N2	160 (8.1%)	113 (8.1%)	47 (7.9%)
N3	13 (0.7%)	7 (0.5%)	6 (1.0%)
**M stage**			
M0	1,816 (91.8%)	1,270 (91.6%)	546 (92.2%)
M1	163 (8.2%)	117 (8.4%)	46 (7.8%)
**Surgery**			
No	90 (4.5%)	68 (4.9%)	22 (3.7%)
Yes	1,889 (95.5%)	1,319 (95.1%)	570 (96.3%)
**Radiotherapy**			
No	1,899 (96.0%)	1,324 (95.5%)	575 (97.5%)
Yes	80 (4.0%)	63 (4.5%)	17 (2.9%)
**Chemotherapy**			
No	1,527 (77.2%)	1,067 (76.9%)	460 (77.7%)
Yes	452 (4.0%)	320 (23.1%)	132 (22.3%)
**Bone metastasis**			
No	1,925 (97.3%)	1,346 (97.0%)	579 (97.8%)
Yes	54 (2.7%)	41 (3.0%)	13 (2.2%)
**Brain metastasis**			
No	1,976 (99.8%)	1,385 (99.9%)	591 (99.8%)
Yes	3 (0.2%)	2 (0.1%)	1 (0.2%)
**Liver metastasis**			
No	1,928 (97.4%)	1,351 (97.4%)	577 (97.5%)
Yes	51 (2.6%)	36 (2.6%)	15 (2.5%)
**Lung metastasis**			
No	1,912 (96.6%)	1,335 (96.3%)	577 (97.5%)
Yes	67 (3.4%)	52 (3.7%)	15 (2.5%)

*TCC, Transitional cell carcinoma; AJCC, American Joint Committee on Cancer*.

### Screening Prognostic Factors for Renal Pelvic TCC Patients

The univariate Cox proportional hazards regression was used to screen prognostic factors, and the results showed that age, tumor size, grade, histologic type, AJCC TNM stage, surgery, radiotherapy, chemotherapy, bone metastasis, brain metastasis, liver metastasis and lung metastasis were OS- and CSS-related factors (Tables 2, 3). Then, all OS- or CSS-related factors were incorporated into the multivariate Cox analysis, and age, tumor size, histologic type, AJCC stage, surgery, chemotherapy, bone metastasis and liver metastasis were determined as independent OS- and CSS-related factors (Tables 2, 3).

**Table 2:** Univariate and multivariate Cox analysis of overall survival in patients with renal pelvic TCC.

	**Univariate analysis**		**Multivariate analysis**	
	**HR (95% CI)**	***P***	**HR (95% CI)**	***P***
**Age, years**				
<71				
71–83	1.546 (1.297–1.842)	<0.001	1.536 (1.283–1.839)	<0.001
>83	2.681 (2.153–3.337)	<0.001	2.578 (2.055–3.235)	<0.001
**Tumor size, mm**				
<45.0				
45.0–67.0	1.752 (1.456–2.109)	<0.001	1.305 (1.078–1.581)	0.006
>67.0	3.176 (2.618–3.852)	<0.001	1.752 (1.415–2.169)	<0.001
**Race**				
White				
Black	0.984 (0.627–1.545)	0.944		
Other	1.025 (0.709–1.480)	0.897		
**Sex**				
Female				
Male	0.929 (0.794–1.087)	0.359		
**Grade**				
I				
II	0.774 (0.422–1.420)	0.408		
III	1.924 (1.112–3.331)	0.019		
IV	1.928 (1.130–3.288)	0.016		
**Histological type**				
TCC, NOS				
Papillary TCC	0.481 (0.411–0.564)	<0.001	0.791 (0.667–0.938)	0.007
**AJCC**				
I				
II	1.680 (1.191–2.370)	0.003	1.766 (1.248–2.498)	0.001
III	2.304 (1.804–2.944)	<0.001	2.174 (1.693–2.791)	<0.001
IV	7.033 (5.540–8.929)	<0.001	5.191 (3.922–6.870)	<0.001
**T stage**				
T1				
T2	1.581 (1.159–2.157)	0.004		
T3	2.353 (1.896–2.920)	<0.001		
T4	7.766 (6.058–9.956)	<0.001		
**N stage**				
N0				
N1	3.254 (2.605–4.064)	<0.001		
N2	3.033 (2.394–3.843)	<0.001		
N3	4.885 (2.178–10.953)	<0.001		
**M stage**				
M0				
M1	5.931 (4.780–7.360)	<0.001		
**Surgery**				
No				
Yes	0.294 (0.222–0.389)	<0.001	0.505 (0.375-−0.681)	<0.001
**Radiotherapy**				
No				
Yes	2.303 (1.704–3.112)	<0.001		
**Chemotherapy**				
No				
Yes	1.368 (1.143–1.638)	0.001	0.670 (0.545–0.822)	<0.001
**Bone metastasis**				
No				
Yes	7.245 (5.155–10.183)	<0.001	2.408 (1.649–3.515)	<0.001
**Brain metastasis**				
No				
Yes	5.995 (1.491–24.103)	0.012		
**Liver metastasis**				
No				
Yes	10.497 (7.362–14.965)	<0.001	2.934 (1.993–4.318)	<0.001
**Lung metastasis**				
No				
Yes	5.587 (4.141–7.538)	<0.001		

*TCC, Transitional cell carcinoma; HR, hazard ratio; CI, confidence interval; AJCC, American Joint Committee on Cancer*.

**Table 3:** Univariate and multivariate Cox analysis of cancer-specific survival in patients with renal pelvic TCC.

	**Univariate analysis**		**Multivariate analysis**	
	**HR (95% CI)**	***P*** **value**	**HR (95% CI)**	***P*** **value**
**Age, years**				
<68				
68–83	1.495 (1.223–1.828)	<0.001	1.509 (1.228–1.853)	<0.001
>83	2.447 (1.891–3.168)	<0.001	2.422 (1.852–3.169)	<0.001
**Tumor size, mm**				
<45.0				
45.0–67.0	2.110 (1.714–2.599)	<0.001	1.508 (1.217–1.870)	<0.001
>67.0	3.882 (3.133–4.811)	<0.001	1.958 (1.545–2.481)	<0.001
**Race**				
White				
Black	1.029 (0.636–1.665)	0.907		
Other	0.944 (0.635–1.401)	0.773		
**Sex**				
Female				
Male	0.975 (0.818–1.162)	0.774		
**Grade**				
I				
II	1.012 (0.465–2.206)	0.976		
III	2.749 (1.343–5.629)	0.006		
IV	2.728 (1.351–5.508)	0.005		
**Histological type**				
TCC, NOS				
Papillary TCC	0.435 (0.364–0.520)	<0.001	0.779 (0.643–0.943)	0.010
**AJCC**				
I				
II	2.170 (1.416–3.326)	<0.001	2.261 (1.471–3.476)	<0.001
III	3.283 (2.398–4.496)	<0.001	3.053 (2.218–4.201)	<0.001
IV	11.171 (8.238–15.149)	<0.001	7.728 (5.475–10.908)	<0.001
**T stage**				
T1				
T2	1.906 (1.319–2.754)	0.001		
T3	3.011 (2.314–3.917)	<0.001		
T4	10.660 (7.974–14.252)	<0.001		
**N stage**				
N0				
N1	3.879 (3.069–4.903)	<0.001		
N2	3.423 (2.652–4.420)	<0.001		
N3	6.008 (2.675–13.493)	<0.001		
**M stage**				
M0				
M1	6.904 (5.506–8.658)	<0.001		
**Surgery**				
No				
Yes	0.255 (0.191–0.341)	<0.001	0.466 (0.341–0.637)	<0.001
**Radiotherapy**				
No				
Yes	2.551 (1.858–3.501)	<0.001		
**Chemotherapy**				
No				
Yes	1.569 (1.294–1.903)	<0.001	0.680 (0.545–0.848)	0.001
**Bone metastasis**				
No				
Yes	8.341 (5.892–11.806)	<0.001	2.573 (1.754–3.775)	<0.001
**Brain metastasis**				
No				
Yes	6.967 (1.732–28.035)	0.006		
**Liver metastasis**				
No				
Yes	11.225 (7.737–16.286)	<0.001	2.487 (1.669–3.705)	<0.001
**Lung metastasis**				
No				
Yes	6.434 (4.726–8.760)	<0.001		

*TCC, Transitional cell carcinoma; HR, hazard ratio; CI, confidence interval; AJCC, American Joint Committee on Cancer*.

### Construction and Validation of the Nomograms for OS and CSS

**Figure 2:**
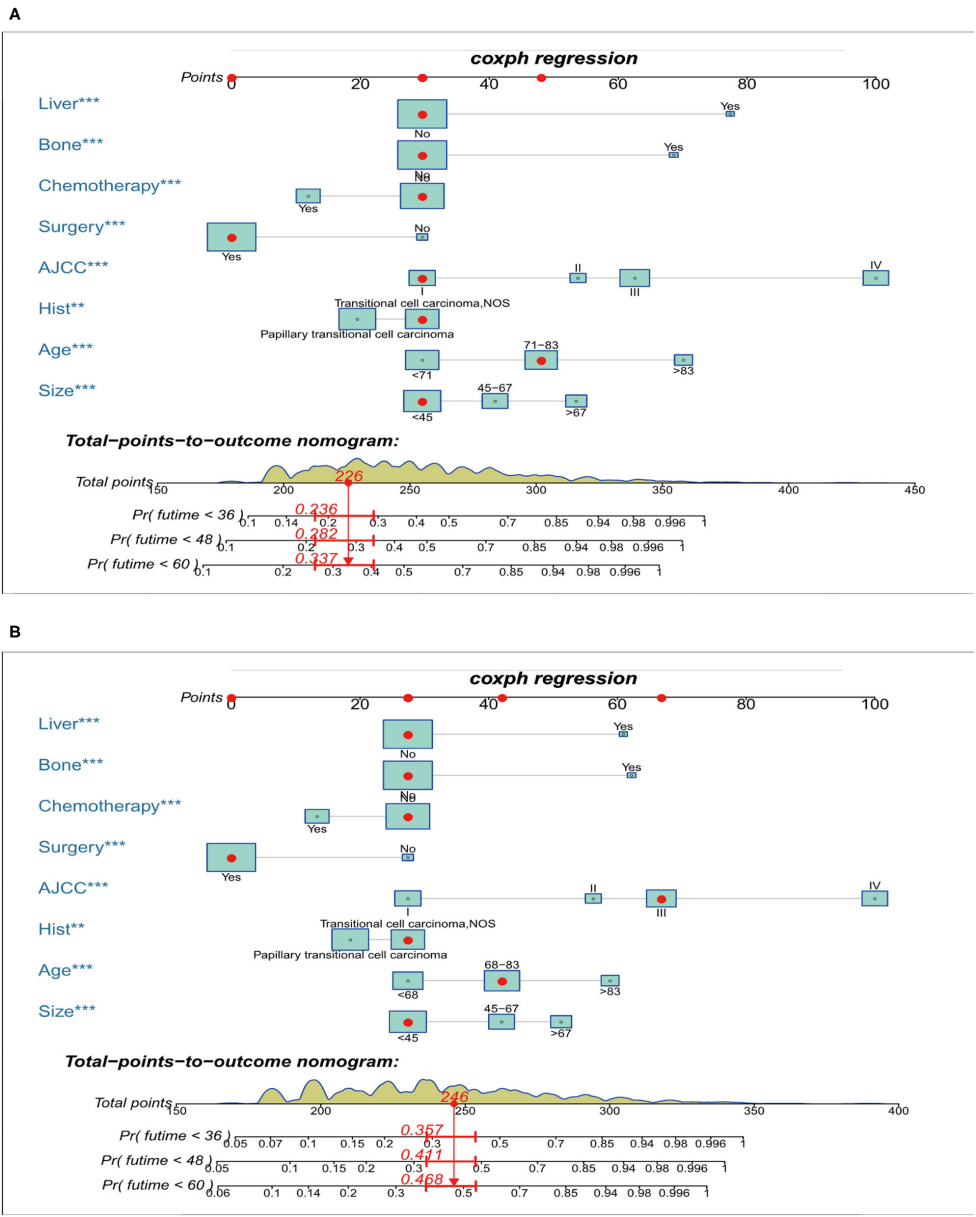
Nomograms for predicting the 3-, 4-, and 5-year overall survival **(A)** and cancer-specific survival **(B)** of patients with renal pelvic TCC. TCC, transitional cell carcinoma; AJCC, American Joint Committee on Cancer; ***P < 0.01, **P < 0.001.

**Figure 3:**
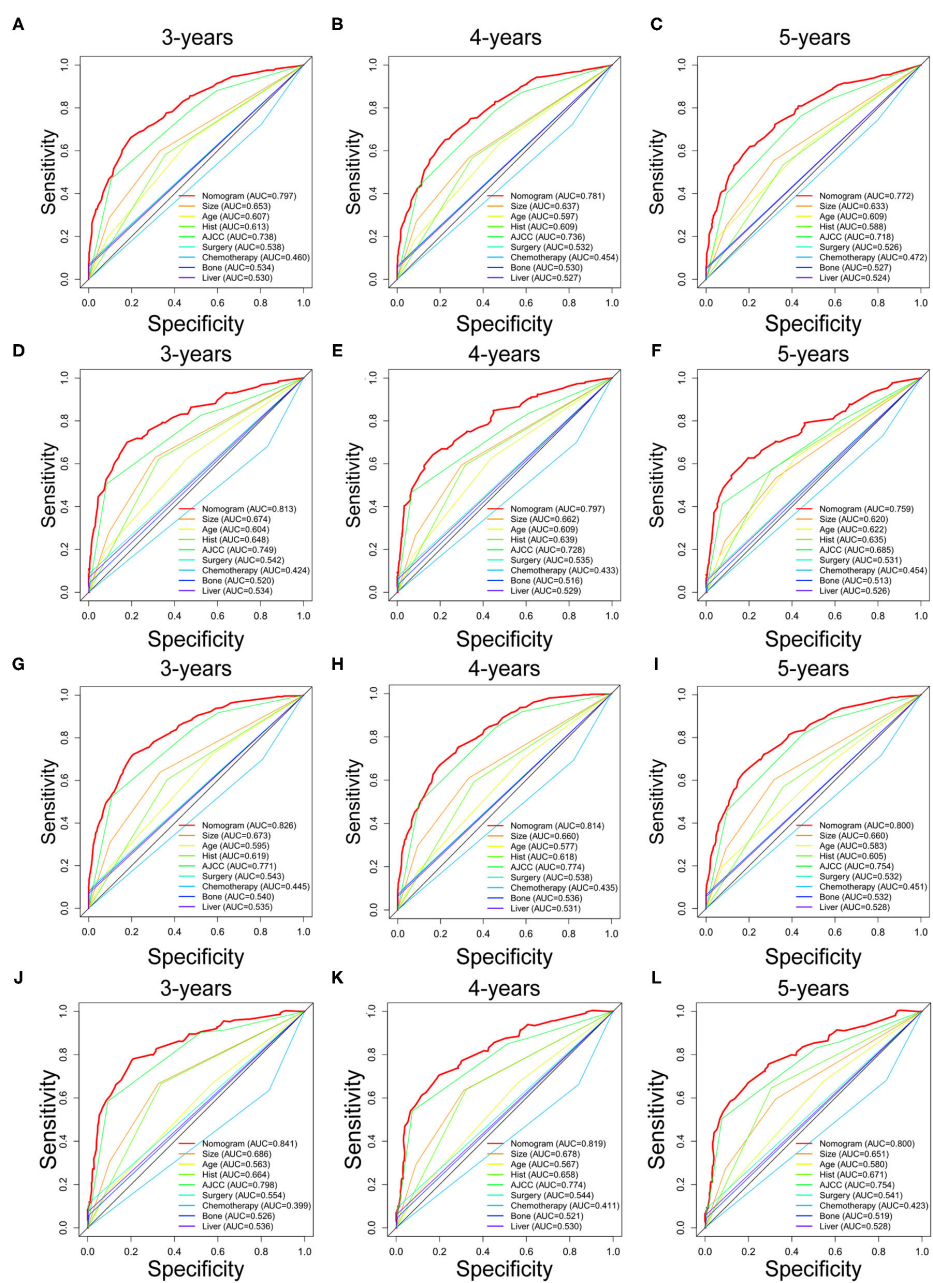
The receiver operating characteristic (ROC) curves of OS nomogram and all independent predictors at 3- **(A)**, 4- **(B)**, and 5-years **(C)** in the training cohort and at 3- **(D)**, 4- **(E)**, and 5-years **(F)** in the validation cohort. The receiver operating characteristic (ROC) curves of CSS nomogram and all independent predictors at 3- **(G)**, 4- **(H)**, and 5-years **(I)** in the training cohort and at 3- **(J)**, 4- **(K)**, and 5-years **(L)** in the validation cohort. OS, overall survival; CSS, cancer-specific survival; AJCC, American Joint Committee on Cancer; AUC, the areas under the ROC curve.

**Figure 4:**
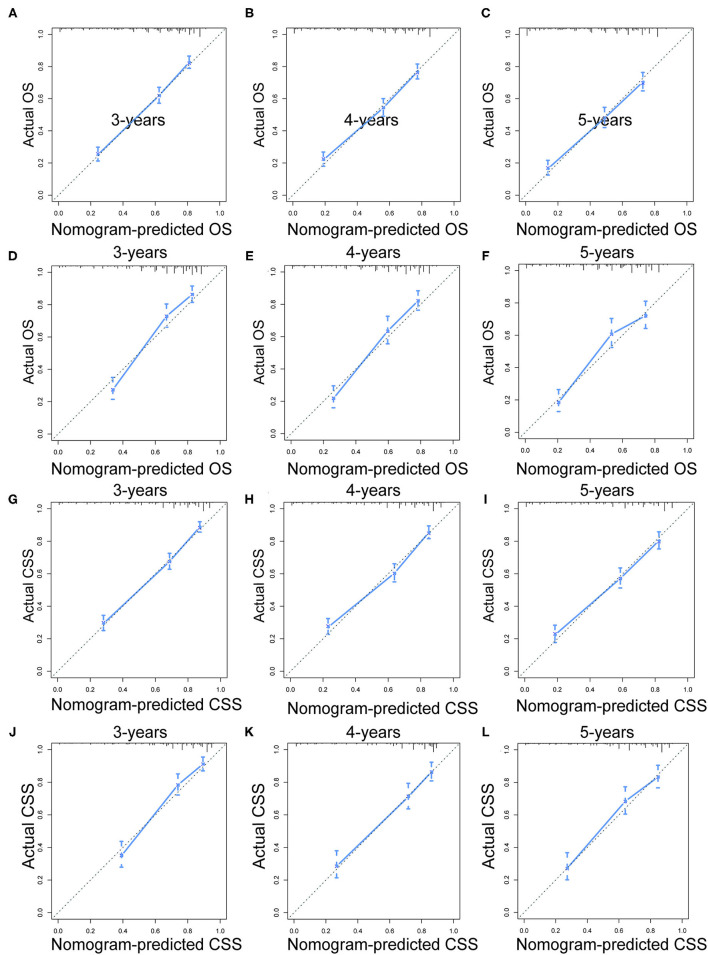
The calibration curves of OS nomogram at 3- **(A)**, 4- **(B)**, and 5-years **(C)** in the training cohort and at 3- **(D)**, 4- **(E)**, and 5-years **(F)** in the validation cohort. The calibration curves of CSS nomogram at 3- **(G)**, 4- **(H)**, and 5-years **(I)** in the training cohort and at 3- **(J)**, 4- **(K)**, and 5-years **(L)** in the validation cohort. OS, overall survival; CSS, cancer-specific survival.

**Figure 5:**
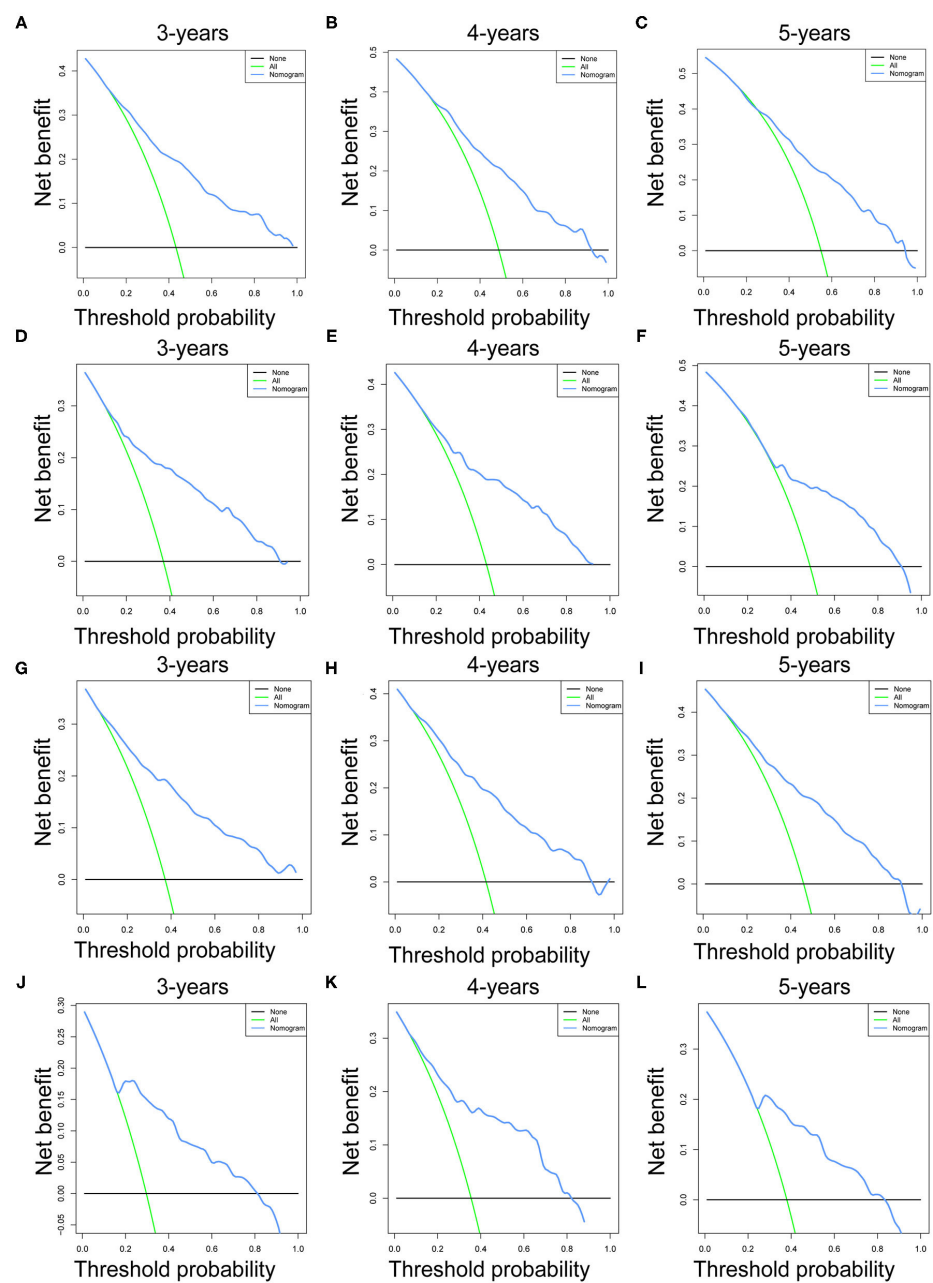
The decision curve analysis (DCA) of OS nomogram at 3- **(A)**, 4- **(B)**, and 5-years **(C)** in the training cohort and at 3- **(D)**, 4- **(E)**, and 5-years **(F)** in the validation cohort. The decision curve analysis (DCA) of CSS nomogram at 3- **(G)**, 4- **(H)**, and 5-years **(I)** in the training cohort and at 3- **(J)**, 4- **(K)**, and 5-years **(L)** in the validation cohort. OS, overall survival; CSS, cancer-specific survival.

Prognostic nomograms of OS and CSS were established by incorporating corresponding independent prognostic factors (Figures 2A,B). For the OS prognostic nomogram, the AUCs in the training cohort at 3-, 4-, and 5-years were 0.797, 0.781, and 0.772, respectively, and the corresponding AUCs were 0.813, 0.797, and 0.759 in the validation cohort (Figures 3A–F). For the CSS prognostic nomogram, the AUCs in the training cohort at 3-, 4-, and 5-years were 0.826, 0.814, and 0.800, respectively, and the corresponding AUCs were 0.841, 0.819, and 0.800 in the validation cohort (Figures 3G–L). Additionally, both in the training cohort and validation cohort, the calibration curves for the probability of 3-, 4-, and 5-years OS and CSS indicated a good consistency between nomogram-predicted OS and CSS and the actual outcome (Figure 4). The DCA curves showed that the nomograms had a good predictive efficiency for OS and CSS of patients with renal pelvic TCC (Figure 5).

### Comparison of Discrimination Between Nomograms and Independent Prognostic Factors

To further show the superior discrimination of our nomograms in predicting the prognosis of renal pelvic TCC patients, we also generated the ROC curves of all independent prognostic factors. The results showed that the AUCs of all prognostic factors alone were higher than 0.500, which means that all individual factors can serve as a reliable prognostic factor. Among them, AJCC stage has the largest AUCs, indicating that AJCC stage is the most effective single indicator. However, the AUCs of all prognostic factors were lower than the AUCs of nomograms, including OS and CSS nomogram (Figure 3). Generally, we confirmed that the discrimination of two nomograms were superior to all the independent prognostic factors.

### Performance of the Nomograms in Stratifying Risk of Patients

**Figure 6:**
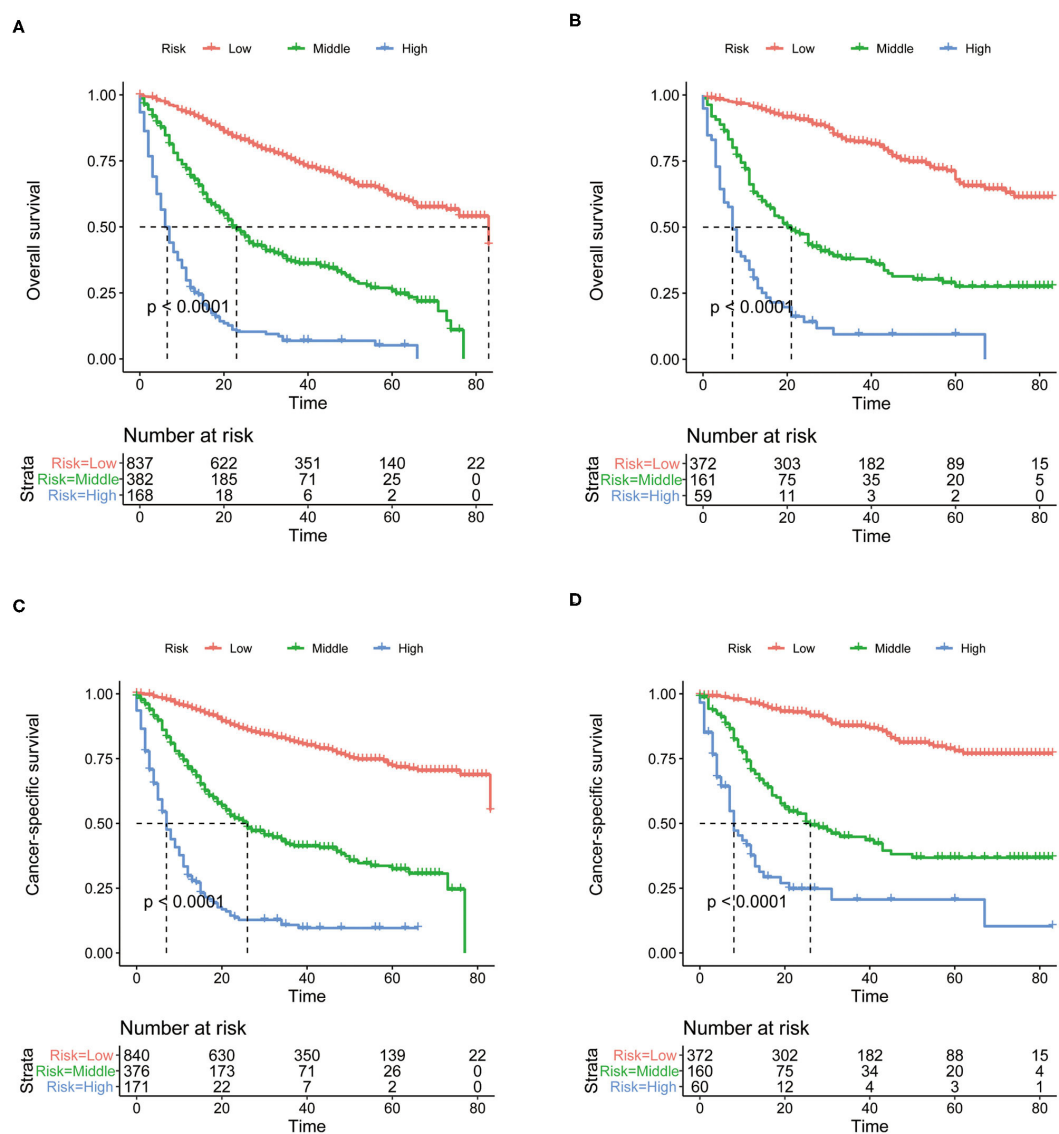
The Kaplan-Meier (K-M) curves of OS nomogram in the training cohort **(A)** and in the validation cohort **(B)**. The Kaplan-Meier (K-M) curves of CSS nomogram in the training cohort **(C)** and in the validation cohort **(D)**. OS, overall survival; CSS, cancer-specific survival.

The total prognostic scores of all patients were calculated by the nomograms. Then, the X-tile software were performed in the training cohort and all patients were divided into low-, middle- and high-risk groups. The K-M curves suggested that patients in the high-risk group had a worse prognosis than those in the middle- and low-risk groups (Figure 6). The cutoff values determined in the training cohort were used in the validation cohort. The prognosis of the three risk groups is significantly different (*P* < 0.0001). Generally, our risk stratification system is very effective.

### Subgroup Analysis to Evaluate the Value of Nomograms

**Figure 7:**
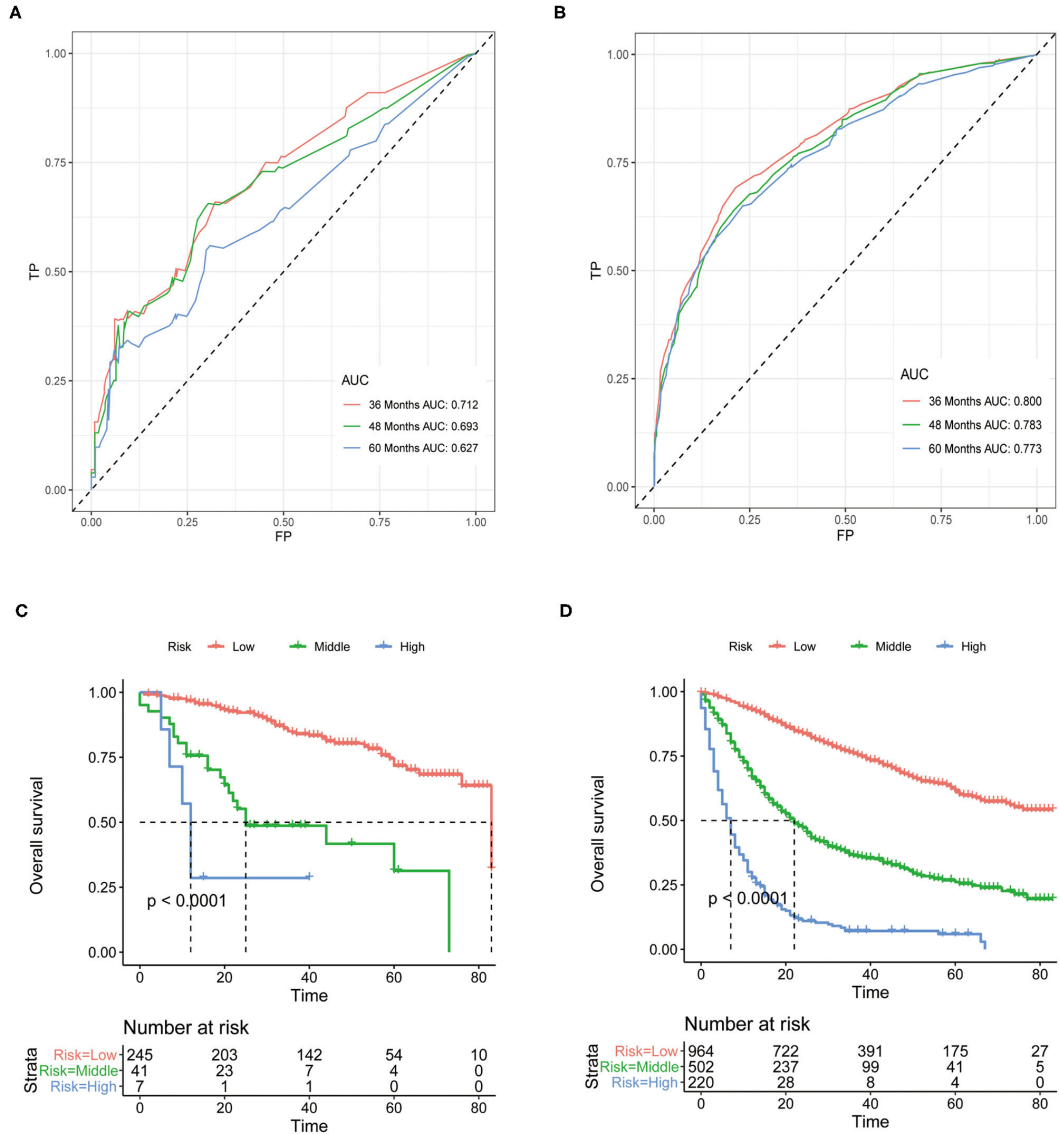
**(A)** The areas under the ROC curves (AUCs) of OS for well differentiated group; **(B)** The areas under the ROC curves (AUCs) of OS for poorly differentiated group; **(C)** The Kaplan-Meier (K-M) curves of OS for well differentiated group (all log-rank *P* values for trend < 0.0001); **(D)** The Kaplan-Meier (K-M) curves of OS for poorly differentiated group (all log-rank *P* values for trend < 0.0001). OS, overall survival.

**Figure 8:**
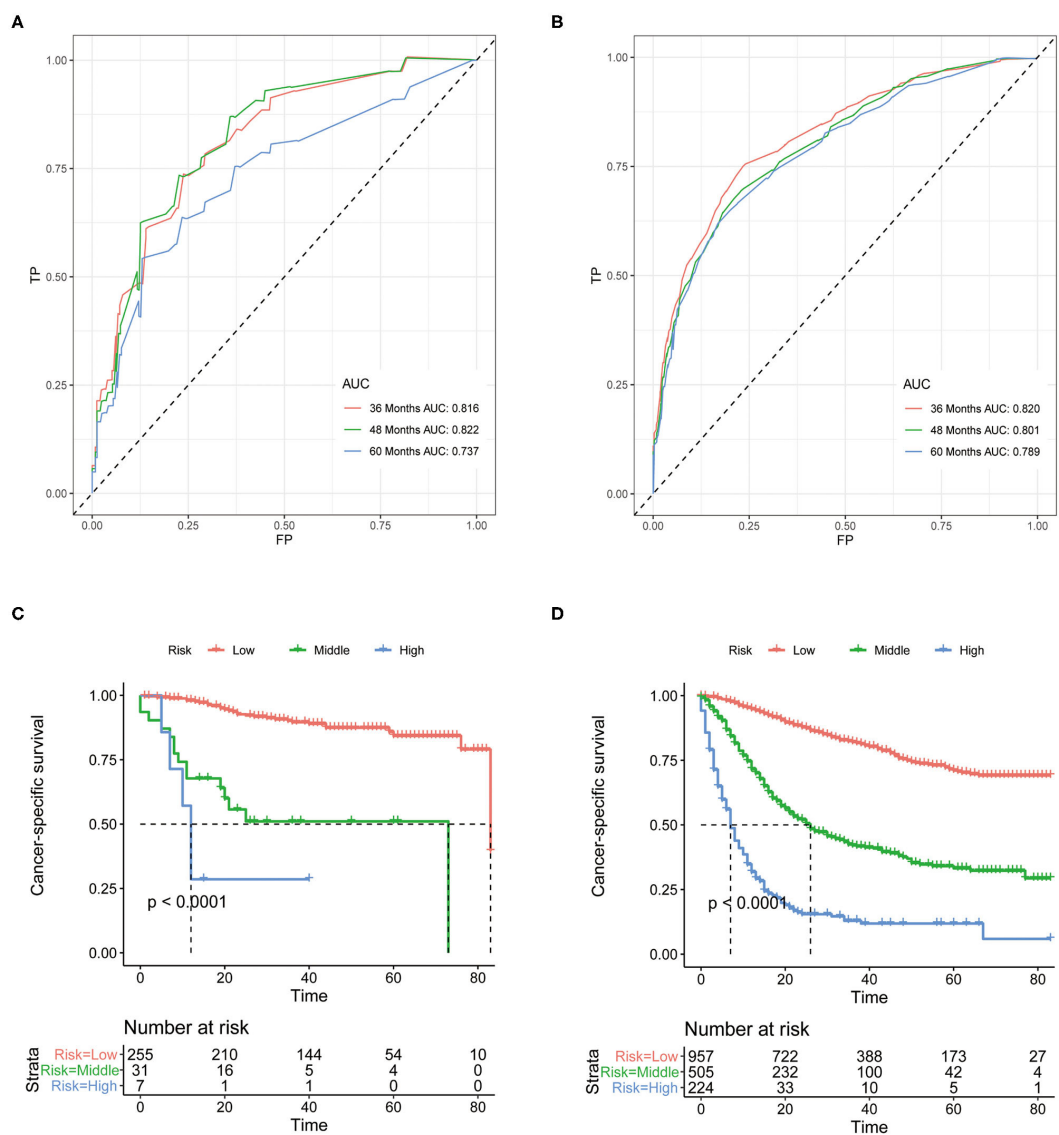
**(A)** The areas under the ROC curves (AUCs) of CSS for well differentiated group; **(B)** The areas under the ROC curves (AUCs) of CSS for poorly differentiated group; **(C)** The Kaplan-Meier (K-M) curves of CSS for well differentiated group (all log-rank *P* values for trend < 0.0001); **(D)** The Kaplan-Meier (K-M) curves of CSS for poorly differentiated group (all log-rank *P* values for trend < 0.0001). CSS, cancer-specific survival.

Although above analyses indicated that both nomograms showed favorable predictive performance, their performance in different patients is still unclear. We defined grade I-II as well differentiated and grade III-IV as poorly differentiated. In the well differentiated group, the AUCs of OS nomogram at 3-, 4-, and 5-years were 0.712, 0.693, 0.627, respectively, and the corresponding AUCs were 0.800, 0.783, and 0.773, respectively, in the poorly differentiated group (Figures 7A,B). The AUCs of CSS nomogram in the well differentiated group at 3-, 4-, and 5-years were 0.816, 0.822, and 0.737, respectively, and the corresponding AUCs were 0.820, 0.801, and 0.789 in the poorly differentiated group (Figures 8A,B). The nomograms showed fairly effective efficiency to discriminate outcomes. Further analysis in the well differentiated group and poorly differentiated group showed that the nomograms were also able to stratify each grade into three significant prognostic groups with low-, middle-, and high-risks of CSS and OS, respectively (Figures 7C,D, 8C,D). Obviously, there were significant prognostic differences between the three groups. Generally, these confirmed robust utility of nomograms in both risk classification and stratification.

## Discussion

Genitourinary cancer remains a major public health problem, and their incidence and mortality rates have changed significantly in recent decades (27). Therefore, there is an urgent need to develop effective predictive tools to guide cancer prevention and treatment. Due to the low incidence of renal pelvic TCC, no prognostic model was developed to predict the prognosis of these patients. In this study, we successfully established and validated nomograms for predicting the prognosis of patients with renal pelvic TCC. Eight indicators were included in the prognostic nomograms of OS and CSS, including age, tumor size, histologic type, AJCC stage, surgery, chemotherapy, bone metastasis, and liver metastasis. Both of these nomograms performed well in predicting the survival of renal pelvic TCC patients. More importantly, the risk stratification systems were constructed, which can not only predict the prognosis, but also guide treatment selection for renal pelvic TCC patients.

Multivariate analysis showed the influence of age on the prognosis of renal pelvic TCC patients. Based on the X-tile software, the age cutoff value of a specific group of people can better determine, in order to better study the impact of age on patient prognosis. As a clinical indicator not considered by the AJCC stage system, age is closely related to the prognosis of cancer patients, including urinary system tumors (28). This may be because the poor prognosis of elderly patients was not only related to the clinical course, but also related to comorbidities (29–31). In addition, taking into account their comprehensive physiological functions, only less active treatment was performed, resulting in a relatively poor prognosis (32). Similarly, this study also determined the specific cutoff value of tumor size for renal pelvic TCC patients. Previous studies on the effect of tumor size on prognosis have reported different findings. In a single-center study, tumor size had no significant effect on survival (33). Matsui et al. (34) and Pieras et al. (35) reported that tumor size was associated with the risk of bladder recurrence. Our study observed a significant correlation between larger tumor size and shorter OS and CSS in the multivariate analysis. It may be because the larger the tumor size, the stronger the aggressive biological characteristics it exhibits, and the worse the condition tends to be.

In our study, AJCC stage was confirmed as a strong predictor in patients with renal pelvic TCC, including OS and CSS. AJCC stage is a widely accepted prognostic factor for cancer patients (36). It considers the primary tumor, local metastasis, and distant metastasis. A number of studies had shown that by integrating AJCC stage and other clinical prognostic indicators, the accuracy of predicting the prognosis of cancer patients can be significantly improved (37, 38). In fact, the current AJCC stage system is mostly formulated for one system of tumors, and lacks a histological or site-specific stage system. Therefore, histological or site-specific nomograms can be used as a supplementary tool to more accurately predict the prognosis of patients.

Interestingly, our study incorporates histological type into the prediction model of renal pelvic TCC. Its biggest advantage is that it can be quickly obtained through ureteroscopy (39). The influence of the histological type of renal pelvic TCC on the prognosis is still controversial. Junichiro performed a retrospective study and found that the papillary structure is associated with recurrence in the bladder (40). Conversely, some scholars had reported that compared with other histological types, the papillary structure is associated with a lower recurrence rate and a higher survival rate (39, 41). Although the papillary structure showed better prognosis in our research, further study is needed.

As for distant disease, we found that patients with distant metastases had worse survival. Consistent with Shinagare et al. (42), liver metastasis and bone metastasis were associated with poor prognosis of UTUC patients. Additionally, Cheaib et al. (43) also revealed that in high-grade UTUC, liver and bone recurrence is relatively quick compared with other sites, and the prognosis is poor. This may be because the urothelium of the ureter and the renal pelvic have the same embryonic origin, leading to similar biological behavior of these epithelial tumors (44). Therefore, it should be considered in the treatment of advanced patients to improve the survival rate of these patients.

Regarding treatment factors, surgery and chemotherapy were independent prognostic factors for renal pelvic TCC patients. Radical nephroureterectomy (RNU) with the bladder cutoff removal is still the standard treatment method for upper urinary tract tumors (45). However, for low-stage and grade patients, The EAU guidelines recommend kidney sparing surgery as the main treatment method (46). In these patients, the survival rate of this method is equivalent to that of radical treatment, and it can reduce the incidence of dysfunction after radical operation (47). The high recurrence rate is one of the important reasons for the poor prognosis of patients with renal pelvic TCC. For advanced patients, although they have been cured by surgery, cancer recurrence should be prevented. Chemotherapy had been proven to inhibit or kill tumor cells to a certain extent, delay tumor recurrence and prolong survival time (48, 49). A large number of retrospective studies had confirmed the survival benefits of adjuvant chemotherapy after surgery (18, 50). Therefore, for patients with a high risk of potential disease recurrence, adjuvant chemotherapy should be considered to prevent cancer recurrence. In the further study, efforts to identify optimal candidates for chemotherapy among renal pelvic TCC patients received surgery is important.

Although the nomogram models performed good accuracy, inevitably, there are some limitations to our work. First, the SEER database lacks some potentially important factors, such as lymphatic vascular invasion, socioeconomic status, comorbidities and other factors related to prognosis. In addition, the SEER database does not distinguish between adjuvant chemotherapy and neoadjuvant chemotherapy. Besides, the SEER database does not provide comprehensive health information, such as specific surgery information. Finally, the nomograms need to be verified in an external cohort before it can be formally used in clinical practice. Therefore, it is necessary to further calibrate the nomogram in the future.

The direct application of our two prognostic nomograms is to predict the prognosis of patients with renal pelvic TCC, including OS and CSS. The risk stratification system directly shows the clinical value of the nomogram. The poorly differentiated and well differentiated groups were further divided into high-, middle- and low-risk groups, which can provide references for the selection and optimization of treatment plans.

## Conclusion

In conclusion, we used routine clinical data to construct and validate the nomograms of patients with renal pelvic TCC at 3-, 4-, and 5- years. The nomogram scoring systems had better discriminative power and clinical application value compared with the prognostic factors alone. Besides, the results of the subgroup analysis of well and poorly differentiation groups confirmed the powerful role of nomograms in distinguishing results and risk stratification. This is very useful for promoting individualized therapy and management of patients with renal pelvic TCC.

## Data Availability Statement

The original contributions presented in the study are included in the article/supplementary material, further inquiries can be directed to the corresponding author/s.

## Ethics Statement

Ethical review and approval was not required for the study on human participants in accordance with the local legislation and institutional requirements. Written informed consent for participation was not required for this study in accordance with the national legislation and the institutional requirements.

## Author Contributions

TH conceived and designed the study, conducted data analysis, and drafted the manuscript. SY contributed with a critical revision of the manuscript. Both authors have read and approved the final version of the manuscript.

## Conflict of Interest

The authors declare that the research was conducted in the absence of any commercial or financial relationships that could be construed as a potential conflict of interest.

## Publisher's Note

All claims expressed in this article are solely those of the authors and do not necessarily represent those of their affiliated organizations, or those of the publisher, the editors and the reviewers. Any product that may be evaluated in this article, or claim that may be made by its manufacturer, is not guaranteed or endorsed by the publisher.

## Abbreviations

TCC: transitional cell carcinoma
OS: overall survival
CSS: cancer-specific survival
SEER, Surveillance, Epidemiology: and End Result
K-M: Kaplan-Meier
ROC: receiver operating characteristic
DCA: decision curve analysis
AJCC: American Joint Committee on Cancer
AUC: area under the curve
UTUC: upper tract urothelial carcinoma
HR: hazard ratio
CI: confidence interval
RNU: radical nephroureterectomy.
